# Evaluation of pre-analytical errors using six sigma metrics towards quality of laboratory performance in an Indian tertiary care hospital

**DOI:** 10.6026/9732063002001418

**Published:** 2024-10-31

**Authors:** Shruti Seth, Narendra Kumar Sah, Neha Rajwal

**Affiliations:** 1 Department of Biochemistry, Shri Mata Vaishno Devi Institute of Medical Excellence and Associated Hospital, Shri Mata Vaishno Devi University, Kakryal, Katra, Jammu and Kashmir 182320, India; 2 Laboratory Medicine, Shri Mata Vaishno Devi Narayana, Superspeciality Hospital, Kakryal, Katra, Jammu and Kashmir, 182320, India; 3 Department of Biochemistry, Maharishi Markandeshwar Medical College and Hospital, Lado, Kumarhatti - Solan 173229 India

**Keywords:** Pre-analytical errors, six sigma metrics, laboratory quality, total laboratory testing, laboratory errors, quality indicators

## Abstract

Six Sigma methodologies is one of the standard management system adopted to check the quality and improve laboratory performance continuously by assessing the laboratory process using six sigma metrics. A one-year prospective study from January 2019 to December 2019 was conducted at the laboratory of medicine department in a tertiary care hospital. A total of 162974 samples were received from both inpatient and outpatient departments and total laboratory errors were found to be 1144, with the maximum errors identified in the pre-analytical phase coming out to be 978 (0.6%). The types of errors in the pre-analytical phase after being identified and categorized were hemolyzed samples, clotted samples, phlebotomy errors, etc. The most typical error identified was clotted sample error (0.29%) followed by haemolysed sample error (0.20%). After calculating the error rate or defects per million error samples on a Sigma calculator, the value obtained was between 4 and 5 which an average class quality. Thus, we show that continuous and repeated recognition of errors and evaluating them using Six Sigma metrics can help achieve overall laboratory procedure quality and better clinical diagnosis.

## Background:

Total Laboratory testing (TTP) is a process that starts with raising a test request of an individual by a clinician, followed by sample collection at the laboratory end for testing and ending with test reports of that individual after testing [1]. As laboratory results play a crucial role in disease diagnosis, it is vital to guarantee the reliability of the results furnished by the clinical laboratory [2]. Errors in this process can disprove the test results concerning the patient's health status [1]. According to many studies, 70% of medical decisions rely on the accuracy of laboratory tests and the whole laboratory testing cycle has three phases: Pre-analytical, Analytical and Post-analytical [3-4]. Over the years, the considerable changes leading to advancements in automation, sample collection, transport and dispatch of results have led to a substantial change in the working and quality of performance in the laboratories with a decline in the errors to a remarkable level [2]. These advancements have provided enormous help in clinical decision-making by supporting, preventing, diagnosing and therapeutic monitoring of human disorders [5]. However, achieving 100% accuracy and precision is far beyond the approachability [2]. Any defect from ordering tests to reporting results and appropriately interpreting and reacting to these defects is known as Laboratory error. These errors, which arise at any phase, can lead to misdiagnosis, mistreatment, *etc.,* [5]. A remarkable decrease is seen in the analytical phase due to the advanced instrument technology compared to the pre-analytical phase error [6], which is reported to be around 46 - 68%, according to some studies [4]. Some other studies showed that 61.9% of errors arose in the pre-analytical phase, while 15% of errors were in the analytical phase and 23.1% were in the post-analytical phase [6]. As evident, the laboratory bears the maximum burden of errors arising in the pre-analytical phase; its regulation and continuous monitoring arise because of unmanageable pre-analytical variables such as too many medical professionals involved, including physicians, nursing, transport staff, phlebotomists, *etc.* is crucial [3]. Therefore, it is of much concern to identify the viable areas of errors in this phase and take corrective steps at repeated intervals to reduce them and, in turn, provide high-quality, reliable test results and safe health care [6]. The areas of errors identified further need to be checked on quality by measuring the defects based on the Six Sigma scale. Six Sigma methodologies were introduced into the industry and business as early as the 1980s and were developed by Motorola, Inc. This methodology measures the error rate or defects expressed as defects per million (DPM) in the pre-analytical phase. It thus monitors the outcome process by converting the errors or defects to sigma metrics using a standard table available in any six-sigma text. Laboratory processes with poor outcomes are counted as errors. Higher value validates the good quality laboratory report results. Six Sigma metrics from 6.0-3.0 represent the range from "best to worst case quality. Six Sigma is a world-class quality process, with the 4 Sigma value considered the average class quality performance [2, 7]. The objective of the present study is to enumerate the types, estimate the frequency and evaluate the quality of the identified pre-analytical phase errors observed during the 1- year study period in the Department of Laboratory Medicine.

## Materials and Methods:

The present descriptive study was conducted in the central Laboratory, Department of Laboratory Medicine, Shri Mata Vaishno Devi Narayana Super Specialty Hospital and a tertiary Care Hospital. A total of 162974 samples were received randomly and 761955 tests were performed from both outpatient and inpatient departments for pre-defined pre-analytical errors during the 1-year duration from January 2019 to December 2019.Phlebotomists collected outpatient samples and inpatient samples were collected by nursing staff and transported to the laboratory by supporting staff. At the sample receiving desk, the person assigned for sample receiving checks for errors and makes entries in the sample rejection register as per standard operating procedure. This data was evaluated monthly using percentages and Six Sigma scale methodology for laboratory testing performance quality checks.

## Six sigma values were calculated by first calculating the DPM rate using the following formula:

DPM = number of errors x 1,000,000/total number of samples

After calculating DPM, the rate was converted to a sigma value based on the sigma score calculators available online at http://www.westgard.com/six-sigma-calculators.htm.

Depending upon the Six Sigma value, the performance level of the laboratory in the pre-analytical phase was evaluated.

## Results:

The present study was conducted in the Central Laboratory during the 1- year duration from January 2019-December 2019. A total of 162974 samples were received and 761955 tests were performed from both outpatient and inpatient departments. Out of 162974 samples received, as per the LIS system of the hospital, the total number of samples found to have laboratory errors is 1144 (0.7%), with 978 (0.6%) errors identified in the pre-analytical phase along with 150 (0.10%) and 16 (0.01%) in both analytical and post-analytical phases (Figure 1 see PDF).

The hospital's LIS system (HINAI) made it easy to trace the sample right from the start, *i.e.,* from when the clinician ordered the test until receiving it. The errors identified and included as quality indicators for the improvement of total laboratory quality were:-

[1] Hemolysed sample

[2] Clotted sample

[3] Phlebotomy error

[4] Wrong sample

[5] Wrong billing

[6] Lipemic

[7] Wrong collection

Month-wise percentage data of pre-analytical errors per error (Figure 2 see PDF) and their Six Sigma value (Figure 3 see PDF) were calculated and significant changes were seen in the month of July (12.2% and 4.7).

The calculated percentage and Six Sigma value of the quality indicators identified and rejected in our laboratory were 28.8% (4.5) of hemolysed samples and 33.2% (4.4) were clotted due to improper mixing or delayed transport. 13.1% (4.7) errors were due to incorrect phlebotomy, 8.7% & 8.4% (4.8 each) samples were rejected because of wrong samples (tube and cap interchange or samples not matching with advised test) and wrong collection (biochemistry sample collected in EDTA tube or vice versa and saliva instead of sputum in case of microbiology tests). 6.24% (4.9) of wrong billing errors were either due to missing tests as requested by the doctor or test requests were wrongly billed. 1.5% (5.3) of lipemic samples was rejected over one year of the study. (Table 1) (Figure 4 see PDF & Figure 5 see PDF). The month-wise distribution of different pre-analytical errors identified is given in Table 2.

## Discussion:

Though the laboratories adopt state-of-the-art facilities, primarily concerned with a fully automated system to give appropriate patient care decisions, there are still laboratory diagnostics errors [8]. The total error rate in the present study was 0.7%, which is in the range of 0.1 - 9.3%, as suggested by Carraro and Plebani for good laboratory care [4]. In the present study, the pre-analytical error was found to be 85.5%, an analytical error was 13% and the post-analytical error was 1.4% concerning total errors. A study on laboratory errors showed that pre-analytical errors were 81% and analytical errors 10%, primarily human and technical errors [9]. In the current location, we first conducted a study pointing out the types and frequencies of pre-analytical errors with a high occurrence of pre-analytical errors in concordance with the previous studies [10, 11]. Most pre-analytical errors were due to clotted samples, hemolysed samples and phlebotomy errors. Hemolysis might be caused by temperature variations, incorrect sample collection (forceful dispensing of the sample through fine needle, vigorous shaking and improper centrifugation), untrained staff and improper storage and transport. It is very pertinent to remind phlebotomists and lab staff that hemolysis is one of the most common causes of pre-analytical errors, causing considerable harm to the accuracy of analytical tests [12, 13]. A similar study done in a laboratory of a tertiary care hospital also showed a higher percentage of hemolyzed sample errors (33.2%) [14]. It is evident from the study's findings that medical and paramedical professionals play a substantial role in the causation of pre-analytical errors, demanding collaboration as key to improving laboratory results quality [15]. Clotted samples accounted for rejection of 0.29% in the present study, possibly due to improper sample mixing with clot activator. This is in accordance with the study done by Arul *et al.* which reported that clotted samples (0.12%) were the second most common error, which might be primarily due to inappropriate mixing of samples after collection [16]. Improper mixing and/or under filled/overfilled EDTA tubes might introduce pre-analytical errors as the latter changed the sample to additive proportion and consequently impacted the results' quality [17, 18].

Wrong sample and collection accounted for a rejection of 0.1% each and wrong billing accounted for a rejection of 0.04% in the present study. A survey by Dudani mentioned that though collecting samples from the wrong patient accounted for a small proportion of sample collection errors, it was a considerable concern for laboratory settings. Wrong billing or data entry non-conformances might arise due to incorrect name and age spelling and incomplete contact information. Inappropriate investigation requests occurred because of unreadable writing on the request slips and deficient medical knowledge linked with insufficient training [19]. Laboratory experts should conduct sensitizing programs for treating clinicians and urge the bedside phlebotomist to take care of the appropriate filling of test request forms to help laboratory workers better validate. Adopting computerized test requests by clinicians, the lab lean process and introducing Six Sigma rules could reduce the frequency of pre-analytical errors [20]. Again, lipemic samples accounted for rejection in the present study was 0.01%. In a study done by Chawla *et al.* it was reported that the rejection of samples due to lipemia was 0.03% and 0.11% in the admitted patients and outpatients, respectively [5]. Lipemia samples might be due to interference of heavy metals, or a patient might suffer from hyperlipoproteinemia disorder. It became mandatory for both the clinician and phlebotomist to ensure that proper patient preparation should be introduced before sample collection [21]. However, lipemia could be avoided by taking overnight fasting samples [5]. Several studies also reported that the significant interference of lipemia in the laboratory analysis and ultracentrifugation procedure might help reduce lipemia in samples [22, 23]. In the present study, we calculated the Six Sigma value for 1-year study and different pre-analytical variables. We did our best in this quality check by adopting Six Sigma metrics of value 4.0 as average class quality performance and less than 3.0 as unsatisfactory. As our Six Sigma values of most of the parameters were between 4 - 5 sigma, which is average class quality, there is a necessity for rigorous quality checks in a pre-analytical zone by identifying and taking care of the gaps in the pre-analytical stage and eventually refining the quality and performance of clinical laboratory by achieving world-class performance of 6.0 sigma value. Various studies adopted Six Sigma Metrics to point out pre-analytical errors and improve the quality and performance of the laboratory [24, 25]. Training the laboratory technicians, nursing staff and interns frequently on sample collection, transport and appropriate filling of test request forms with all required information is necessary. Making mistakes is human nature and those who correct the errors are good humans [26].

## Conclusion:

The quality of laboratory performance for identifying, categorizing the pre-analytical errors as quality indicators for interpreting the Six Sigma value is of concern. This process helped us take remedial actions needed like training technicians, nurses and doctors to avoid and reduce the number of errors for eventually improving the total quality management in the laboratory.

## Aim:

The main aim of our study is to identify the pre-analytical errors and laboratory quality assessment based on these errors using the Six Sigma methodology.

## Abbreviations:

EDTA = Ethylene diamine tetraacetic acid

TTP = Total testing process

DPM = Defects per million

LIS = Laboratory Information System

HINAI = Software of LIS

## Figures and Tables

**Table 1:** The various pre-analytical errors observed in the present study were summarized as follows:

**S. No.**	**Type of Pre-analytical error(s)**	**Frequency**
1	Clotted samples	325 (0.29%)
2	Hemolysed samples	282 (0.20%)
3	Phlebotomy error	128 (0.18%)
4	Wrong sample	85 (0.10%)
5	Wrong Collection	82 (0.10%)
6	Wrong billing	61 (0.04%)
7	Lipemic samples	15 (0.01%)

**Table 2:** Further, the distribution of different pre-analytical errors month-wise was presented as follows:

**Type of Pre-analytical Errors**	**Month**											
	**Jan**	**Feb**	**Mar**	**Apr**	**May**	**Jun**	**Jul**	**Aug**	**Sep**	**Oct**	**Nov**	**Dec**
HEMOLYSED	24	24	29	22	22	23	35	29	21	26	11	16
CLOTTED	29	11	28	27	27	32	48	3	31	17	36	36
PHLEBOTOMY ERROR	15	22	9	16	3	5	12	14	10	19	1	2
WRONG SAMPLE	4	17	1	2	2	7	11	5	6	8	7	15
WRONG BILLING	6	7	6	0	0	1	1	5	10	11	10	4
LIPEMIC	2	2	2	1	1	2	0	1	0	2	0	2
WRONG COLLECTION	7	5	7	4	4	5	12	11	5	9	10	3
